# Lesion feature enhancement and boundary-aware fusion for pulmonary embolism recognition on CT images

**DOI:** 10.3389/fmed.2026.1846588

**Published:** 2026-06-18

**Authors:** Jinbiao Li

**Affiliations:** The First People's Hospital of Neijiang, Neijiang, China

**Keywords:** boundary aware fusion, CT images, deep learning, feature enhancement, pulmonary embolism

## Abstract

**Objective:**

To address the challenges of complex lesion representation, blurred vascular boundaries, and insufficient model generalization ability in pulmonary embolism CT images, an automatic recognition method that combines lesion feature enhancement and boundary-related structural cue fusion is proposed.

**Methods:**

An end to end pulmonary embolism recognition framework was constructed. On the basis of hierarchical visual encoding, a Pulmonary Embolus Feature Enhancement Module and a Vascular Boundary Aware Fusion Module were introduced to strengthen lesion discriminative representation and feature-level boundary-related structural modeling, respectively. Comparative experiments, ablation studies, visualization analysis, and external dataset validation were conducted to systematically evaluate the effectiveness of the proposed method in the binary classification setting.

**Results:**

On 523 single-center chest CT cases, the proposed method achieved an Accuracy of 0.956, a Precision of 0.961, a Recall of 0.951, and an AUC of 0.971, outperforming multiple comparison models in the internal evaluation. In external validation, the proposed method achieved Accuracy values of 0.779 and 0.672 on the FUMPE and RSNA datasets, respectively, indicating its potential cross-domain generalization ability in pulmonary embolism recognition.

**Conclusion:**

The proposed method can effectively improve the discriminative performance of binary pulmonary embolism recognition from CT images, and may provide a useful technical reference for computer-aided screening of pulmonary embolism.

## 1 Introduction

Pulmonary embolism is a serious cardiopulmonary vascular disease caused by emboli obstructing the main trunk of the pulmonary artery or its branches. It is characterized by sudden onset, rapid progression, and a relatively high mortality rate, and is one of the most critical acute diseases threatening patient survival in clinical practice (1, 2). Because its clinical manifestations are often nonspecific, patients may present with a variety of nonspecific symptoms such as chest pain, dyspnea, and tachycardia. As a result, it is easily confused with other respiratory or cardiovascular diseases during actual diagnosis and treatment, thereby increasing the risks of misdiagnosis and missed diagnosis (3). Meanwhile, the global mortality burden related to pulmonary embolism remains substantial and has become one of the important factors affecting public health (4). In this context, CT pulmonary angiography, as a key imaging examination method in the current diagnosis of pulmonary embolism, can directly reflect intrapulmonary arterial filling defects and related anatomical structural changes, thereby providing an important basis for the recognition and evaluation of pulmonary embolism (5, 6).

In recent years, with the rapid development of artificial intelligence and medical image analysis technologies, research on automatic pulmonary embolism recognition based on CTPA has continued to deepen. Soffer et al. systematically summarized the application progress of deep learning in pulmonary embolism detection, indicating that this direction has become an important research hotspot in intelligent medical image analysis (7). Ma et al. further introduced a multitask learning mechanism into pulmonary embolism detection and recognition tasks in order to enhance the model's ability to jointly model complex imaging signs (8). Kim et al. developed and validated a non-contrast CT-based pulmonary embolism detection framework using GAN-generated synthetic contrast enhancement, further demonstrating the potential value of image enhancement and contrast-aware representation learning for pulmonary embolism analysis (9). In studies oriented toward clinical application, Wiklund et al. explored the value of deep learning algorithms in the detection and triage of cancer associated incidental pulmonary embolism, whereas Ayobi et al. analyzed the role of artificial intelligence tools in pulmonary embolism detection workflows from the perspectives of performance and clinical practicality (10, 11). In addition, Bushra et al. proposed a dual pathway deep learning framework for pulmonary embolism detection on CTPA, Abdelhamid et al. attempted to combine a hybrid vision transformer with deep learning techniques to improve pulmonary embolism recognition performance, and Hassan et al. summarized the overall development status of current artificial intelligence methods for pulmonary embolism detection on CTPA from the perspective of a systematic review (12–14).

Although related studies have made certain progress, intelligent pulmonary embolism recognition from CT images still faces several key challenges. First, pulmonary embolism lesions in CTPA images often present complex signs such as local filling defects, blurred boundaries, and subtle grayscale differences, which makes it difficult for models to achieve fine grained lesion representation and stable discrimination (15). Second, the experimental settings and data sources in existing studies vary greatly, and the actual performance and reliability of deep learning models still fluctuate considerably across different studies, indicating that this field still lacks sufficiently consistent and robust validation conclusions (16). In addition, although some methods have achieved favorable results on specific datasets, their generalization ability under cross center, cross protocol, and external independent data conditions remains limited, which to some extent restricts their further clinical deployment and promotion (17).

To address the above issues, this study focused on the task of automatic pulmonary embolism recognition from CT images and proposed a deep learning method that combines lesion feature enhancement and vascular boundary aware fusion mechanisms. Based on a hierarchical visual representation learning framework, this study designed a Pulmonary Embolus Feature Enhancement Module to strengthen cross level interaction of lesion related features and their discriminative representation. At the same time, a Vascular Boundary Aware Fusion Module was constructed to improve the model's perception of pulmonary artery contours, boundary transition regions, and abnormal density variations through the joint modeling of vascular boundary structural information and local texture style information. Furthermore, through comparative experiments, ablation studies, visualization analysis, and external dataset validation, this study systematically evaluated the effectiveness, internal working mechanism, and cross domain generalization ability of the proposed method, thereby providing a solution for intelligent pulmonary embolism recognition from CT images with improved separation between embolism-related filling defects and normal vascular representations and potential utility for computer-aided pulmonary embolism screening.

## 2 Materials and methods

### 2.1 Dataset description and ethical approval

This study focused on the task of pulmonary embolism recognition based on CT images, and the data used in this study were derived from retrospective chest CT imaging records accumulated during previous clinical diagnosis and treatment processes at The First People's Hospital of Neijiang. All cases were obtained from imaging data generated during the hospital's routine examination workflow. Specifically, patients underwent contrast-enhanced CT pulmonary angiography (CTPA) or contrast-enhanced chest CT examinations for suspected or clinically evaluated pulmonary embolism during clinical diagnosis, condition assessment, or related examination needs, and, after ethical approval had been granted, the research team retrospectively organized, screened, and annotated the existing imaging records rather than recruiting additional participants or introducing any new acquisition procedures specifically for this study. A total of 523 cases were included, consisting of 273 normal cases and 250 pulmonary embolism cases. The unit of inclusion and data partitioning was the patient-level examination/case, while the computational input used in the current classification framework was one representative two-dimensional axial CT image selected from each included case. Therefore, each CT sample in this study refers to a representative two-dimensional axial CT image extracted from a patient-level CTPA or contrast-enhanced chest CT examination, rather than the entire three-dimensional CT volume. For pulmonary embolism cases, the representative image was selected from the slice level containing diagnostically relevant pulmonary artery filling defects or embolism-related imaging findings. For normal cases, the representative image was selected from the corresponding pulmonary artery level without pulmonary embolism evidence. The pulmonary embolism category was determined according to the final clinical diagnosis, while the normal category was confirmed based on the corresponding imaging findings and clinical records. Non-contrast CT images and examinations without sufficient diagnostic image quality for pulmonary artery assessment were not included in the current dataset. To more clearly illustrate the data source, composition, acquisition method, and sample definition used in this study, Table 1 systematically summarizes the basic information of the dataset. Based on the above dataset construction strategy, the dataset used in this study could adequately reflect the distribution characteristics of pulmonary embolism and normal chest CT images in a real clinical environment, thereby providing a reliable foundation for subsequent model training and generalization performance evaluation.

**Table 1:** Basic information of the pulmonary embolism CT recognition dataset used in this study.

Item	Description
Research task	Automatic pulmonary embolism recognition based on chest CT images.
Data source	Archived chest CT imaging records accumulated during previous clinical diagnosis and treatment processes at The First People's Hospital of Neijiang.
Imaging modality	Contrast-enhanced CTPA or contrast-enhanced chest CT examinations used for pulmonary embolism assessment. Non-contrast CT images were not included in the current dataset.
Data acquisition method	All images were obtained from the hospital's routine clinical examination workflow. Specifically, patients underwent chest CT scanning during actual clinical visits for diagnostic or evaluative purposes, and the study retrospectively organized and analyzed the existing examination results without introducing any additional acquisition specifically for research purposes.
Acquisition parameters	The examinations were retrospectively collected from routine clinical imaging archives. Scanner type, slice thickness, reconstruction parameters, and contrast protocol may vary across routine examinations, and complete scanner-level metadata were not uniformly available for all cases.
Study design	Single-center retrospective study.
Unit of analysis	The inclusion and data partitioning unit was the patient-level examination/case. The model input was one representative two-dimensional axial CT image selected from each included case.
Sample definition	Each CT sample refers to one representative two-dimensional axial CT image extracted from a patient-level CTPA or contrast-enhanced chest CT examination, rather than a full three-dimensional CT volume.
Image selection criteria	For pulmonary embolism cases, the representative image was selected from the slice level showing diagnostically relevant pulmonary artery filling defects or embolism-related imaging findings. For normal cases, the representative image was selected from the corresponding pulmonary artery level without pulmonary embolism evidence.
Total number of samples	523 cases.
Number of normal samples	273 cases.
Number of pulmonary embolism samples	250 cases.
Pulmonary embolism location	Detailed location stratification, including central, lobar, segmental, and subsegmental emboli, was not completely and consistently available in the retrospective records for all pulmonary embolism cases. Therefore, PE location distribution was not used as a stratification factor in the current analysis.
Label determination method	Category labels were assigned according to the clinical diagnosis results, imaging examination conclusions, and related medical records of the corresponding cases.
Data nature	Anonymized medical imaging data used only for scientific analysis and model development.
Training set usage	Used for model training, parameter learning, and optimization.
Test set usage	Used for final model performance validation and generalization ability evaluation.

Regarding acquisition information, all images were collected retrospectively from routine clinical examinations rather than from a prospectively controlled imaging protocol. Therefore, scanner models, slice thickness, reconstruction kernels, contrast injection parameters, and reconstruction parameters may vary across examinations under the hospital's routine clinical workflow. The available DICOM and clinical archive information was reviewed during data organization, but not all acquisition parameters were completely and uniformly recorded for every retrospective case. Accordingly, the present study reports the available modality and sample selection information and acknowledges the incomplete scanner-level acquisition metadata as a limitation of the retrospective dataset.

Regarding data partitioning, to ensure the standardization of model development and performance validation, all 523 cases were evaluated using a five-fold cross-validation strategy. Specifically, the entire dataset was randomly divided into five mutually exclusive subsets at the case level, and in each fold, four subsets were used for model training and parameter optimization, while the remaining subset was used for performance evaluation. The data partition was performed at the case level, and the representative CT image corresponding to each case was assigned to only one fold, thereby avoiding overlap between the training and testing data across the cross-validation process. This strategy enabled each case to be used once for testing and four times for training across the five validation rounds, thereby providing a more stable and comprehensive assessment of the model's recognition capability on previously unseen samples. The training subsets were mainly used to enable the model to learn the differential imaging patterns between pulmonary embolism-related features and normal anatomical structures, whereas the testing subset in each fold was used to objectively assess the model's recognition capability. It should be emphasized that all data used in this study were retrospective materials that had already been clinically acquired and archived, and the research process did not involve any additional human intervention, supplementary image acquisition, or extra examinations. Therefore, the data acquisition process did not affect the patients' original diagnostic or treatment workflow. This design not only conforms to the general standards of retrospective medical studies, but also improves the consistency between the research results and actual clinical application scenarios.

This study was conducted in strict accordance with the principles of the Declaration of Helsinki and was approved by the Ethics Committee of The First People's Hospital of Neijiang, with the ethical approval number 2025KY144. Since all data used in this study were retrospective imaging records generated during previous clinical examinations, and the implementation of the study did not involve any additional participant recruitment, human intervention, specimen collection, or supplementary examinations, while all data had been anonymized before entering the study, the Ethics Committee approved a waiver of informed consent. This ethical approval and the waiver of informed consent indicate that the present study fully satisfied the fundamental requirements for patient privacy protection, data security management, and ethical compliance in medical research when conducting the task of intelligent pulmonary embolism recognition based on CT images. At the level of ethics and data management, this study only involved the organization, annotation, and modeling analysis of existing clinical imaging data, without altering the patients' original diagnostic and treatment workflow or imposing any additional risk on the patients. All research data were used under controlled conditions and were strictly limited to the scope of this study in order to ensure data security, confidentiality, and traceability. Based on the above ethical standards and principles of data use, this study provides a compliant, clear, and reproducible data foundation for the subsequent construction, evaluation, and application of pulmonary embolism CT recognition models.

### 2.2 Data preprocessing

Since the CT imaging data used in this study had already been organized and screened in advance, no additional image quality screening step was introduced during the data preprocessing stage. During the initial data organization stage, two experienced clinical imaging experts independently reviewed the imaging records, category labels, and representative slice selection results. The consistency of their preliminary judgments was assessed using inter-observer agreement analysis, and cases with inconsistent judgments were further discussed until a consensus was reached. Instead, standardized processing was directly performed according to the input requirements of the model. Specifically, the original CT images were first de-identified to remove sensitive information such as patient names, hospitalization numbers, and examination identifiers, thereby ensuring that the use of the data complied with privacy protection requirements in medical research. Subsequently, the image storage format, matrix size, and grayscale representation were unified to reduce data distribution differences caused by different devices, scanning parameters, and export methods. On this basis, grayscale values were normalized so that different samples had a relatively consistent numerical range before being fed into the model, thereby improving convergence stability and feature learning efficiency during model training. For the task of pulmonary embolism recognition, the core objective of preprocessing was not to alter the lesions themselves, but rather to preserve as much key imaging information as possible regarding the pulmonary arteries and their surrounding anatomical structures, while reducing the interference of irrelevant background information and data heterogeneity in the model discrimination process.

To further illustrate the data preprocessing procedure used in this study, Table 2 summarizes the major processing steps and their corresponding purposes. It should be noted that the preprocessing strategy in this study mainly focused on data unification, numerical normalization, expert consensus verification, and model adaptability, without involving any additional manual quality exclusion or repeated screening. The expert review process was used to confirm the reliability of label assignment and representative image selection rather than to introduce an additional subjective filtering procedure during model preprocessing. After the above processing, all samples were organized into standardized input data that could be directly used for deep learning training and testing, and the same preprocessing strategy was applied to both the training set and the test set to avoid introducing additional bias caused by differences in processing procedures. Through this preprocessing design, the comparability among different samples could be enhanced while preserving pulmonary embolism-related imaging features as much as possible, thus providing a reliable data foundation for the subsequent stable recognition of pulmonary embolism and normal CT images.

**Table 2:** Preprocessing pipeline of CT imaging data and its purpose.

Processing step	Specific operation	Purpose
De-identification	Remove patient names, examination identifiers, hospitalization numbers, and other personally identifiable information, while retaining only the imaging data and labels required for the study.	Protect patient privacy and satisfy ethical and research compliance requirements.
Expert review and consensus verification	Two experienced clinical imaging experts independently reviewed the imaging records, category labels, and representative slice selection results. Inter-observer consistency was assessed before consensus discussion, and inconsistent cases were resolved through joint review.	Improve the reliability of label assignment and representative image selection, and reduce potential uncertainty caused by retrospective data organization.
Image format unification	Convert the original CT data into a unified image storage format and ensure consistency in data reading procedures and model input interfaces.	Reduce differences in storage formats across multi-source data and facilitate batch processing.
Size standardization	Resize CT images with different resolutions or matrix dimensions to a unified size so that they meet the fixed input requirements of the model.	Ensure consistent input dimensions and improve the stability of training and inference.
Grayscale normalization	Normalize the pixel intensity values of CT images so that the grayscale distributions of different samples fall within a relatively stable numerical range.	Reduce grayscale fluctuations caused by different scanning conditions and improve feature learning performance.
Label alignment	Uniformly encode samples into two categories, normal and pulmonary embolism, and establish a one-to-one correspondence between images and labels.	Ensure accurate matching between images and class labels during supervised learning.
Dataset organization	Arrange samples into directory structures according to the training and test split results to ensure standardized data loading and experimental reproducibility.	Improve the standardization and reproducibility of the experimental workflow.

### 2.3 Proposed method

#### 2.3.1 Problem definition

In this study, pulmonary embolism recognition based on chest CT images was formulated as a supervised binary classification task. Let the dataset be denoted as $D={\left\{\left(X_{i},y_{i}\right)\right\}}_{i=1}^{N}$, where *N* represents the total number of samples, $X_{i}\in {\mathbb{R}}^{H\times W\times C}$ denotes the *i*-th CT sample, *H*, *W*, and *C* represent the height, width, and number of channels of the image, respectively, and *y*_*i*_∈{0, 1} denotes the corresponding class label, where *y*_*i*_ = 0 indicates a normal sample and *y*_*i*_ = 1 indicates a pulmonary embolism sample. Given an input image *X*_*i*_, the objective is to learn a mapping function from the image space to the label space, denoted as *f*(·;θ), where θ represents the model parameters, such that the model can output the predicted probability that the sample belongs to pulmonary embolism, expressed as ${\hat{p}}_{i}=f\left(X_{i};\theta \right)$, and further obtain the predicted label as ${ŷ}_{i}=\arg\max_{c\in \left\{0,1\right\}}P\left(y_{i}=c|X_{i};\theta \right)$. During the training stage, the model parameters are optimized by minimizing the classification loss between the ground-truth labels and the prediction results, and the objective function can be written as $L_{cls}=-\frac{1}{N}\overset{N}{\underset{i=1}{\sum }}\left[y_{i}\log\left({\hat{p}}_{i}\right)+\left(1-y_{i}\right)\log\left(1-{\hat{p}}_{i}\right)\right]$. Therefore, the core task of this study is to learn feature representations with good robustness and generalization ability while preserving the discriminative capability for distinguishing normal pulmonary structures from pulmonary embolism lesions, thereby enabling accurate recognition of pulmonary embolism and normal samples in CT images.

#### 2.3.2 Overall model architecture

**Figure 1:**
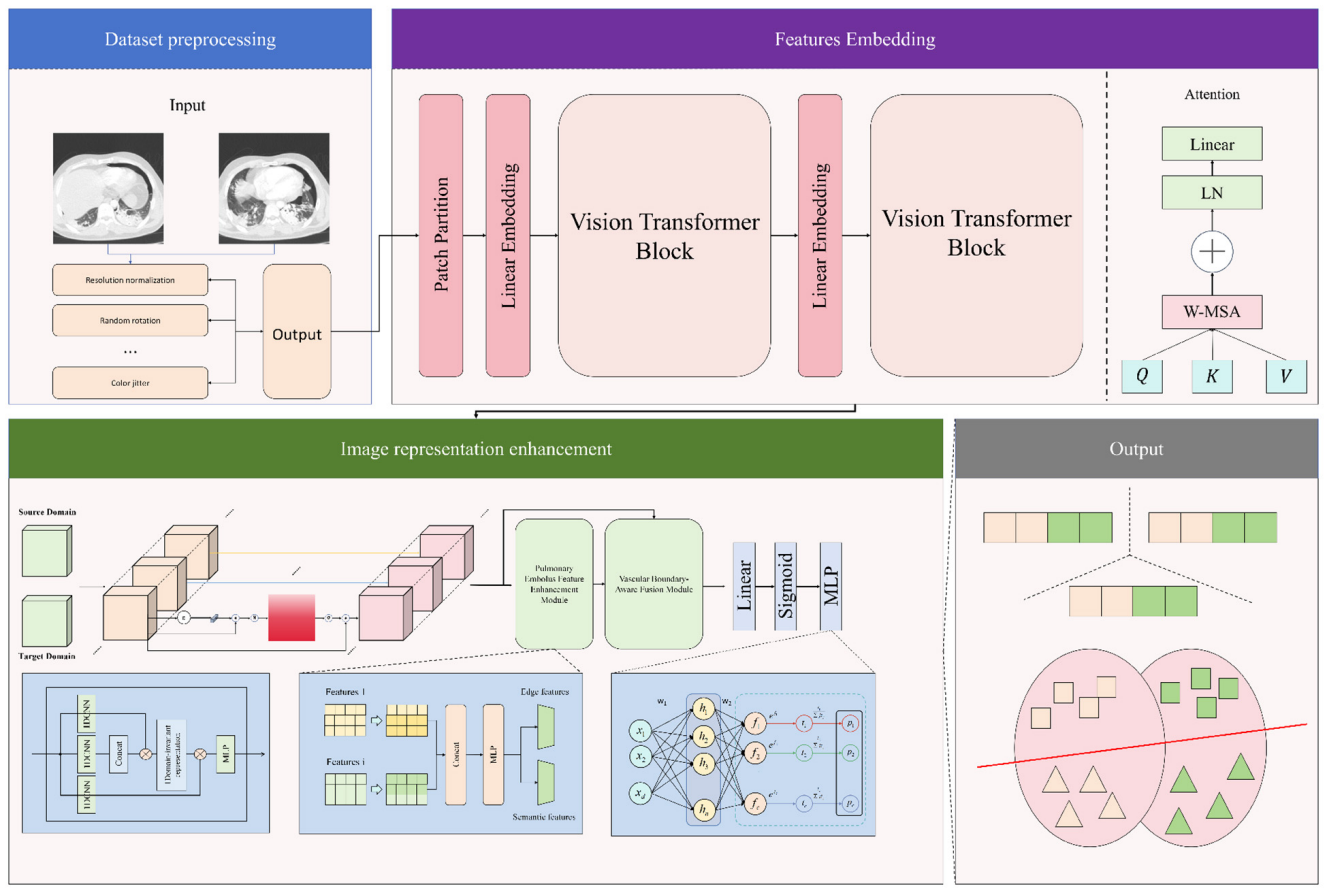
The overall framework of the proposed method for pulmonary embolism recognition from CT images. The model consists of dataset preprocessing, feature embedding, image representation enhancement, and output prediction, where the Pulmonary Embolus Feature Enhancement Module and the Vascular Boundary-Aware Fusion Module are introduced to strengthen lesion-related discriminative representations and vascular boundary-aware semantic modeling.

Based on the above problem formulation, this study constructed an end to end deep network for pulmonary embolism recognition on CT images. The overall pipeline consists of four stages, including data preprocessing, feature embedding, image representation enhancement, and classification output, and its overall architecture is shown in Figure 1.

For an input sample $X_{i}\in {\mathbb{R}}^{H\times W\times C}$, resolution normalization and basic augmentation operations are first applied to obtain the preprocessed result ${\tilde{X}}_{i}$, after which an initial visual token sequence is constructed through image patch partitioning and linear mapping so that the original CT image can be transformed from the pixel space into a feature space suitable for Transformer modeling. This process can be uniformly expressed as shown in Equation 1


$$\begin{array}{l} {\text{Z}}_{i}^{0}=E\left({\tilde{X}}_{i}\right)=L\left(P\left({\tilde{X}}_{i}\right)\right) \end{array}$$ (1)

where P(·) denotes the operation of dividing the input image into several local patches, L(·) denotes the embedding mapping that performs linear projection on each patch, and ${\text{Z}}_{i}^{0}$ denotes the initial feature representation. Subsequently, the model employs a hierarchical visual encoder to perform multi stage contextual modeling on ${\text{Z}}_{i}^{0}$ so as to progressively extract the pulmonary artery region, local density variations, and abnormal embolism related structural representations. In the middle and high level feature spaces, a pulmonary embolism feature enhancement module and a vascular boundary aware fusion module are further introduced to jointly strengthen lesion discriminative information and boundary structural information, thereby improving the model's perception ability for pulmonary embolism signs under complex CT manifestations.

During the overall inference process, the deep features output by the encoder not only contain global contextual semantics, but also further integrate local lesion responses and vascular boundary constraint information. Therefore, the final classification result is jointly determined by the enhanced discriminative features. Let the overall mapping of the backbone network and the two functional modules be denoted as Φ(·), and let the classification head be denoted as C(·). Then, the overall prediction process for an input sample can be expressed as shown in Equation 2


$$\begin{array}{l} {\hat{p}}_{i}=C(\text{Φ}\left({\text{Z}}_{i}^{0}\right))=C(F_{\text{VBF}})F_{\text{PEE}}\left(T\left({\text{Z}}_{i}^{0}\right)\right))) \end{array}$$ (2)

where T(·) denotes the backbone feature encoding process composed of multiple Vision Transformer Blocks, $F_{\text{PEE}}\left(\cdot \right)$ denotes the Pulmonary Embolus Feature Enhancement Module, which is used to strengthen the key response regions associated with pulmonary embolism, $F_{\text{VBF}}\left(\cdot \right)$ denotes the Vascular Boundary Aware Fusion Module, which is used to integrate vascular boundary cues with high level semantic information, and ${\hat{p}}_{i}$ is consistent with the previous definition and represents the predicted probability that sample *X*_*i*_ belongs to the pulmonary embolism category. Based on this overall architecture, the model is able to establish, in a layer by layer manner, a continuous representation pathway from low level textures and local structures to high level lesion discriminative semantics from preprocessed CT images, thereby achieving effective discrimination between normal samples and pulmonary embolism samples.

#### 2.3.3 Pulmonary Embolus Feature Enhancement Module

**Figure 2:**
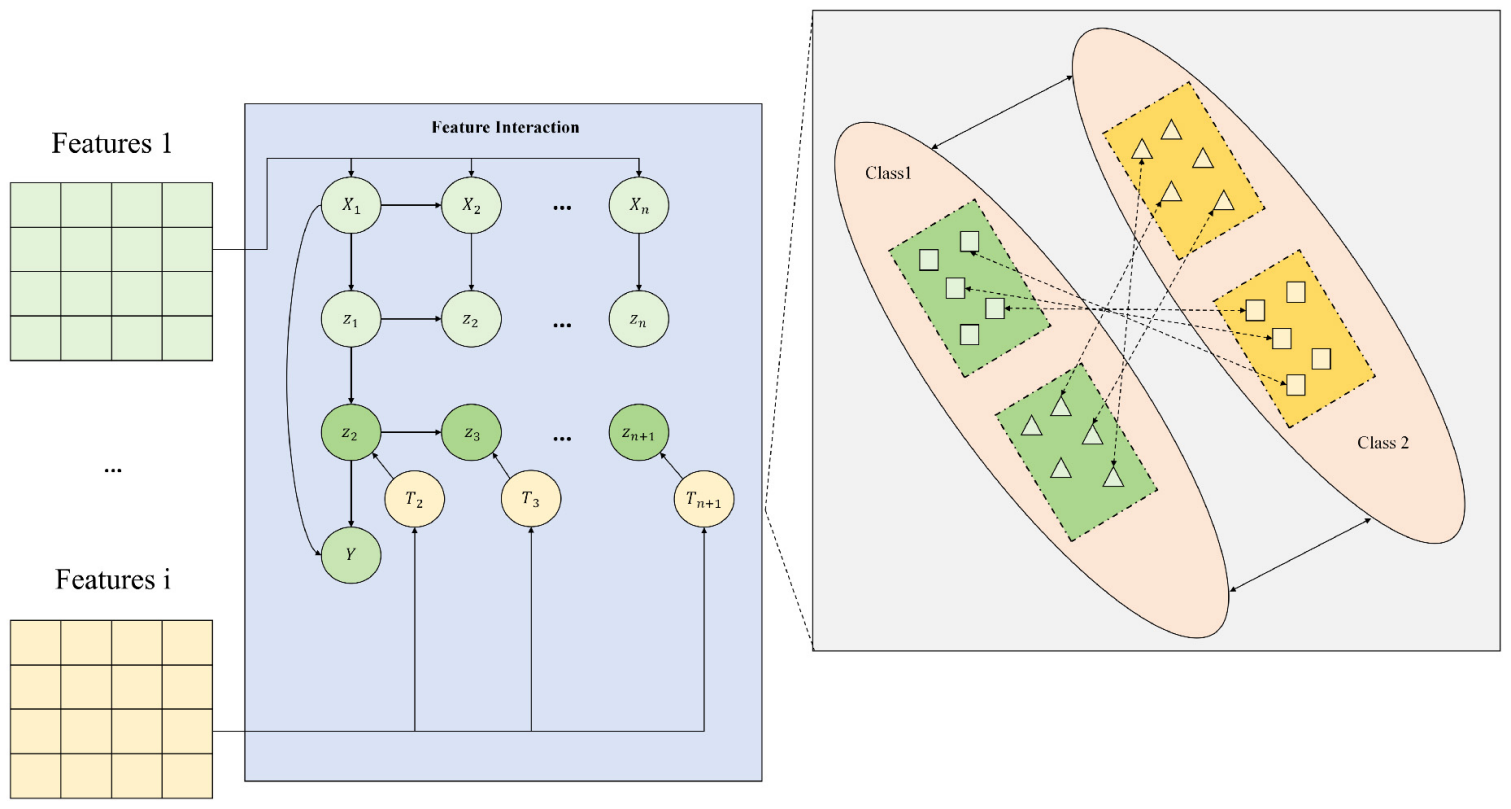
Illustration of the Pulmonary Embolus Feature Enhancement Module, which performs feature-level cross-level feature interaction and adaptive representation enhancement to improve intra-class compactness and inter-class separability for pulmonary embolism recognition.

Within the overall architecture, after the backbone feature encoding process T(·), multi level semantic representations of the input sample *X*_*i*_ can be obtained. Considering that pulmonary embolism in CT images often simultaneously presents multiple signs, including local filling defects, abnormal intravascular density, and changes in neighborhood structural relationships, a single level of features is insufficient to fully describe its discriminative patterns. Therefore, this study designed a Pulmonary Embolus Feature Enhancement Module to perform interactive modeling and collaborative enhancement of features from different levels, and the architecture of this module is shown in Figure 2. Instead of directly concatenating multi level features or applying attention within a single feature layer, the proposed module first aligns features from different stages into a shared representation space and then performs cross level relation propagation with adaptive gated modulation. This design enables lesion related responses distributed across different semantic levels to be selectively enhanced within the classification feature space. The whole process relies only on image level classification labels and does not introduce manual lesion masks, vascular boundary annotations, or segmentation supervision.

Let the feature set from different stages be denoted as ${\left\{{\text{F}}_{i}^{\left(m\right)}\right\}}_{m=1}^{M}$, where ${\text{F}}_{i}^{\left(m\right)}\in {\mathbb{R}}^{N_{m}\times d_{m}}$ represents the feature representation at the *m*th level, *M* denotes the number of feature levels involved in enhancement, and *N*_*m*_ and *d*_*m*_ denote the number of tokens and the channel dimension of the corresponding level, respectively. To facilitate cross level modeling, each level of features is first projected into a shared feature space through a linear mapping, yielding


$$\begin{array}{l} {\text{U}}_{i}^{\left(m\right)}={\text{F}}_{i}^{\left(m\right)}{\text{W}}_{m}+{\text{b}}_{m},\;m=1,2,\ldots ,M \end{array}$$ (3)

As shown in Equation 3 where **W**_*m*_ and **b**_*m*_ denote the learnable projection parameters corresponding to the features of the *m*th level, respectively, and ${\text{U}}_{i}^{\left(m\right)}\in {\mathbb{R}}^{N_{m}\times d}$ denotes the mapped feature with unified dimensionality. Through this operation, the representations of pulmonary artery structures, local textures, and lesion semantics from different levels are aligned into the same space, thereby providing a unified input basis for subsequent cross level interaction and discriminative enhancement.

After obtaining the features in the unified space, this module further constructs a cross level feature interaction mechanism to explicitly model the associations among different levels that are relevant to pulmonary embolism recognition. Specifically, all level features are first concatenated into a node set ${\text{U}}_{i}=\left[{\text{U}}_{i}^{\left(1\right)};{\text{U}}_{i}^{\left(2\right)};\cdots \;;{\text{U}}_{i}^{\left(M\right)}\right]$, and then an interaction adjacency matrix is generated according to feature similarity to characterize the information transmission strength among different local regions and semantic levels, which is defined as shown in Equation 4


$$\begin{array}{l} {\text{A}}_{i}=\text{Softmax}\left(\frac{{\text{U}}_{i}{\text{U}}_{i}^{⊤}}{\sqrt{d}}\right) \end{array}$$ (4)

where **A**_*i*_ denotes the feature interaction matrix of sample *X*_*i*_ in the enhancement module, and each matrix element reflects the degree of correlation between any two feature nodes. On this basis, the interaction enhanced contextual representation is obtained through relation propagation as shown in Equation 5


$$\begin{array}{l} {\text{R}}_{i}={\text{A}}_{i}{\text{U}}_{i} \end{array}$$ (5)

Since pulmonary embolism lesions are often not determined by a single local texture alone, but are jointly related to adjacent vessel walls, intraluminal regions, and cross scale responses, the above interaction propagation process can aggregate complementary information from different levels into the same feature pathway. Furthermore, in order to emphasize the salient responses that are more relevant to pulmonary embolism discrimination, this study introduces a gated enhancement strategy, in which the weighting coefficients are adaptively generated according to the joint state of the original projected features and the interaction features, namely, as shown in Equation 6


$$\begin{array}{l} {\text{G}}_{i}=\sigma \left({\text{W}}_{g}\left[{\text{U}}_{i}||{\text{R}}_{i}\right]+{\text{b}}_{g}\right) \end{array}$$ (6)

where σ(·) denotes the Sigmoid function, [·||·] denotes the feature concatenation operation, and **G**_*i*_ denotes the adaptive modulation weight of each feature node in the enhancement process. This mechanism can effectively suppress redundant responses that are irrelevant to classification while preserving interaction patterns that are closely related to pulmonary embolism signs.

To further improve intra class compactness and inter class separability, the module finally applies the gating weights to the fused representation of the original features and the interaction features, thereby forming the enhanced discriminative feature output for pulmonary embolism. Let the overall mapping of the enhancement module be denoted as $F_{\text{PEE}}\left(\cdot \right)$, then its output can be expressed as shown in Equation 7


$$\begin{array}{l} F_{\text{PEE}}\left({\text{H}}_{i}\right)={\text{G}}_{i}⊙{\text{R}}_{i}+\left(1-{\text{G}}_{i}\right)⊙{\text{U}}_{i} \end{array}$$ (7)

where ${\text{H}}_{i}=T\left({\text{Z}}_{i}^{0}\right)$ is consistent with the previous definition and denotes the high level features output by the backbone network, and ⊙ denotes element wise multiplication. After being processed by this module, the local lesion signals, cross region dependency relationships, and potential class discriminative cues that were originally scattered across different levels are further integrated, enabling normal samples and pulmonary embolism samples to form clearer distribution boundaries in the feature space. As a result, the Pulmonary Embolus Feature Enhancement Module not only realizes effective interaction among multi level features, but also enhances the discriminability of pulmonary embolism related patterns at the level of representation learning, and provides more discriminative and structurally consistent input features for the subsequent Vascular Boundary Aware Fusion Module.

#### 2.3.4 Vascular Boundary-Aware Fusion Module

**Figure 3:**
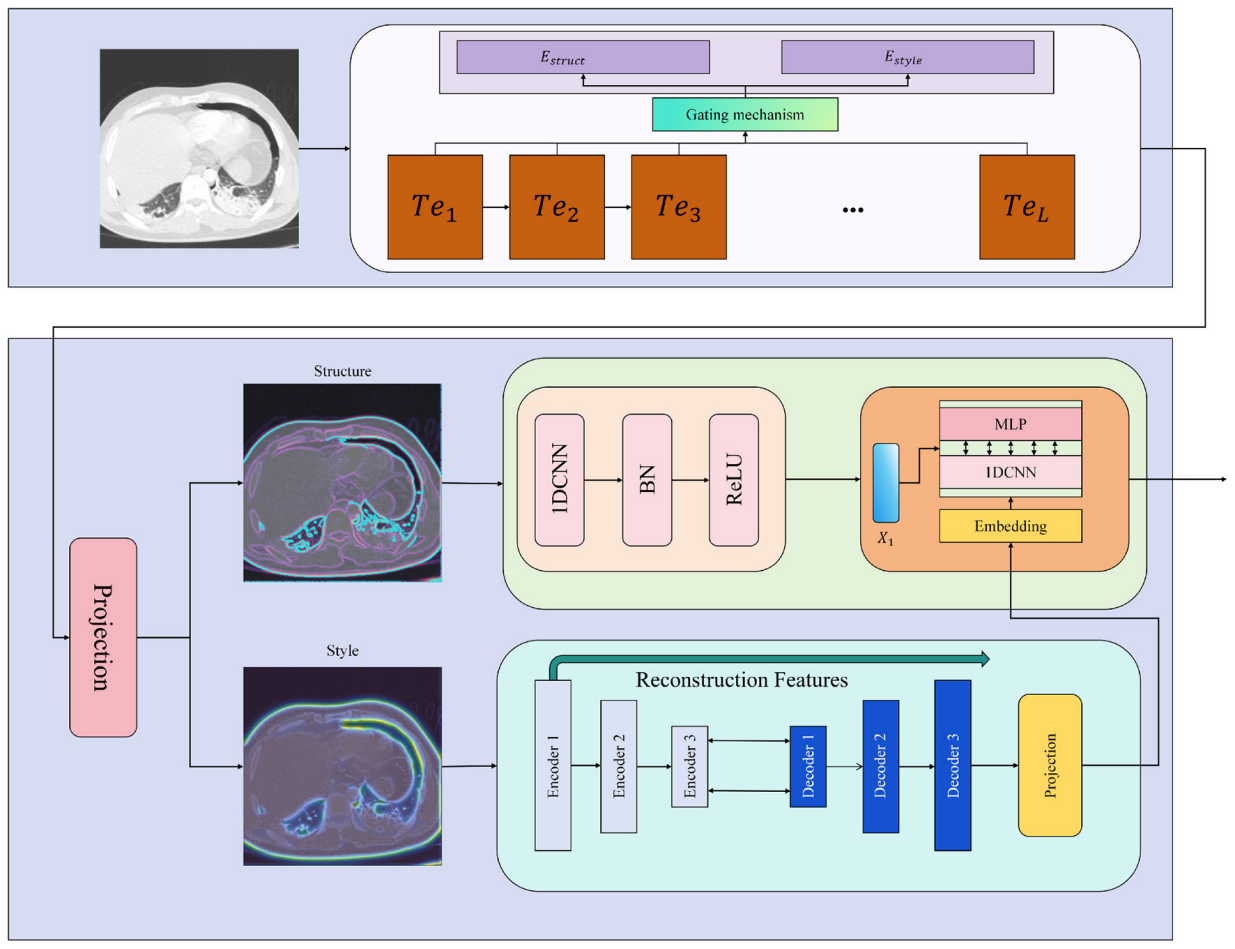
Illustration of the Vascular Boundary-Aware Fusion Module, which jointly models feature-level boundary-related structural cues and style-aware reconstruction information to refine embolism-related representations for pulmonary embolism recognition in CT images.

After being processed by the Pulmonary Embolus Feature Enhancement Module, the enhanced feature $F_{\text{PEE}}\left({\text{H}}_{i}\right)$ has already aggregated cross level semantic interaction information to a considerable extent and improved the intra class compactness and inter class separability of pulmonary embolism related patterns. However, for pulmonary embolism recognition on CT images, relying only on high level discriminative features may still overlook boundary-related structural variations and local density transitions in the feature space, especially when the intraluminal filling defect in the pulmonary artery is small, the boundary is blurred, or the local contrast is low, in which case representation confusion may easily occur between lesion regions and normal vascular structures. Based on this, the present study further designed a Vascular Boundary Aware Fusion Module, which jointly models feature-level structural cues and texture-related cues on the basis of the output of the previous module so as to strengthen the model's classification-oriented representation of boundary-related vascular structures, intraluminal transition patterns, and local abnormal density variations. This module does not use true vascular boundary annotations, pixel-level lesion masks, or segmentation supervision; instead, the term “boundary aware” refers to the use of feature-level transformations to encode boundary-related structural information for image-level classification. The architecture of this module is shown in Figure 3.

Let the output of the previous module be denoted as ${\text{P}}_{i}=F_{\text{PEE}}\left({\text{H}}_{i}\right)$, where ${\text{P}}_{i}\in {\mathbb{R}}^{L\times d}$. It is first decomposed into the structural branch input and the style branch input through a projection operator, that is, as shown in Equation 8


$$\begin{array}{l} \left({\text{P}}_{i}^{str},{\text{P}}_{i}^{sty}\right)=\Pi_{b}\left({\text{P}}_{i}\right) \end{array}$$ (8)

where Π_*b*_(·) denotes the feature projection mapping for boundary aware fusion, and ${\text{P}}_{i}^{str}$ and ${\text{P}}_{i}^{sty}$ denote the structural branch feature and the style branch feature, respectively. This dual branch design maintains continuity with the enhancement module described above, but the focus shifts from global discriminative enhancement to feature-level modeling of boundary-related structural information, thereby forming a progressive processing pathway in the feature stream from lesion discriminative enhancement to boundary aware fusion.

For the structural branch, this study employs a feature-level structural encoding pathway composed of one dimensional convolution, batch normalization, and nonlinear activation to focus on extracting responses associated with vascular contour variations, vessel wall transitions, and local morphological changes in the learned representation space. Its structural boundary embedding is defined as shown in Equation 9


$$\begin{array}{l} {\text{E}}_{i}^{str}=\phi_{b}^{str}\left({\text{P}}_{i}^{str}\right)=\text{ReLU}\left(\text{BN}\left(\text{Conv1D}\left({\text{P}}_{i}^{str}\right)\right)\right) \end{array}$$ (9)

For the style branch, an encoder decoder style reconstruction pathway is used to model grayscale distribution, texture contrast, and local density variation reconstruction at the feature level, so as to characterize the style difference between abnormal perfusion regions within the pulmonary artery lumen and the normal background. Its style reconstruction embedding can be expressed as shown in Equation 10


$$\begin{array}{l} {\text{E}}_{i}^{sty}=\phi_{b}^{sty}\left({\text{P}}_{i}^{sty}\right)=\text{Proj}\left(\text{Dec}\left(\text{Enc}\left({\text{P}}_{i}^{sty}\right)\right)\right) \end{array}$$ (10)

After obtaining these two types of boundary-related feature embeddings, in order to explicitly characterize the complementary relationship between structural information and style information, this study further constructs a boundary fusion representation as shown in Equation 11


$$\begin{array}{l} {\text{B}}_{i}=\psi \left(\left[{\text{E}}_{i}^{str}||{\text{E}}_{i}^{sty}\right]\right) \end{array}$$ (11)

where ψ(·) denotes a fusion function composed of linear mapping and nonlinear transformation, and **B**_*i*_ denotes the jointly encoded boundary aware semantic representation. This representation simultaneously contains information on boundary-related structural variation, local morphological turning, and abnormal density texture, and can therefore enhance the classification representation of pulmonary embolism related patterns on CT images more comprehensively.

To enable the boundary-related feature information to adaptively act on the enhanced discriminative features from the previous stage, this study further introduces a gated fusion mechanism, in which the boundary modulation weights are generated according to the joint state of the structural branch and the style branch. Specifically, the gating vector is defined as shown in Equation 12


$$\begin{array}{l} {\text{M}}_{i}=\sigma \left({\text{W}}_{m}{\text{B}}_{i}+{\text{b}}_{m}\right) \end{array}$$ (12)

where σ(·) denotes the Sigmoid function, **W**_*m*_ and **b**_*m*_ denote learnable parameters, respectively, and **M**_*i*_ denotes the response strength of different feature dimensions to boundary-related information. Then, this gating weight is applied to the joint mapping of the preceding enhanced feature and the boundary fusion representation, yielding the final boundary aware fusion output as shown in Equation 13


$$\begin{array}{l} F_{\text{VBF}}\left({\text{P}}_{i}\right)={\text{M}}_{i}⊙{\text{P}}_{i}+\left(1-{\text{M}}_{i}\right)⊙{\text{W}}_{f}{\text{B}}_{i} \end{array}$$ (13)

where **W**_*f*_ denotes the linear mapping matrix used for dimensional alignment, and ⊙ denotes element wise multiplication. Furthermore, to maintain consistency with the output form of the overall model, the final classification probability can be written as shown in Equation 14


$$\begin{array}{l} {\hat{p}}_{i}=C\left(F_{\text{VBF}}\left({\text{P}}_{i}\right)\right)=C\left(F_{\text{VBF}}\left(F_{\text{PEE}}\left({\text{H}}_{i}\right)\right)\right) \end{array}$$ (14)

where C(·) denotes the classification head, and ${\hat{p}}_{i}$ is consistent with the previous definition and represents the predicted probability that sample *X*_*i*_ belongs to the pulmonary embolism category. In this way, the Vascular Boundary Aware Fusion Module and the Pulmonary Embolus Feature Enhancement Module form a closely connected functional relationship. The Pulmonary Embolus Feature Enhancement Module strengthens cross level discriminative interaction, whereas the Vascular Boundary Aware Fusion Module further refines the enhanced representation by incorporating feature-level boundary-related structural and texture information. Thereby, the model can improve the separation between normal vascular representations and pulmonary embolism related abnormal representations under complex CT scenarios, without performing explicit vascular boundary segmentation or embolism localization.

### 2.4 Evaluation metrics

To comprehensively evaluate the classification performance of the proposed model on the task of pulmonary embolism recognition from CT images, this study selected Accuracy, Precision, Recall, Specificity, F1-score, and the Area Under the ROC Curve, denoted as AUC, as the evaluation metrics. Among them, Accuracy was used to measure the overall prediction consistency of the model on all samples, Precision was used to reflect the proportion of samples that were truly pulmonary embolism among those predicted as pulmonary embolism, Recall was used to describe the detection ability of the model for true pulmonary embolism samples, Specificity was used to evaluate the model's ability to correctly identify normal cases and control false positive predictions, F1-score was used to provide a balanced evaluation of Precision and Recall, and AUC was used to comprehensively evaluate the capability of the model to distinguish normal samples from pulmonary embolism samples under different decision thresholds. Let the true positive, true negative, false positive, and false negative be denoted by *TP*, *TN*, *FP*, and *FN*, respectively. Then, Accuracy is defined as shown in Equation 15:


$$\begin{array}{l} \text{Acc}=\frac{TP+TN}{TP+TN+FP+FN} \end{array}$$ (15)

This metric reflects the consistency between the model predictions and the ground truth labels from an overall perspective. However, in medical classification tasks, relying solely on Accuracy may be insufficient to fully characterize the recognition performance for positive and negative samples, and therefore additional metrics are required for comprehensive evaluation.

Precision is used to measure the proportion of truly positive samples among those predicted as positive by the model, and it is defined as shown in Equation 16:


$$\begin{array}{l} \text{Precision}=\frac{TP}{TP+FP} \end{array}$$ (16)

Recall is used to measure the proportion of all true positive samples that are successfully identified by the model, and it is defined as shown in Equation 17:


$$\begin{array}{l} \text{Recall}=\frac{TP}{TP+FN} \end{array}$$ (17)

Specificity is used to measure the proportion of true negative samples that are correctly identified by the model, and it is defined as shown in Equation 18:


$$\begin{array}{l} \text{Specificity}=\frac{TN}{TN+FP} \end{array}$$ (18)

F1-score is the harmonic mean of Precision and Recall, which reflects the balance between false positive control and positive case detection. It is defined as shown in Equation 19:


$$\begin{array}{l} \text{F1}=\frac{2\times \text{Precision}\times \text{Recall}}{\text{Precision}+\text{Recall}} \end{array}$$ (19)

For the task of pulmonary embolism recognition, Precision reflects the false alarm control ability of the model, whereas Recall directly reflects the detection ability for pulmonary embolism cases. Specificity further characterizes the recognition reliability for normal cases, which is important for reducing unnecessary alerts in computer-aided screening. F1-score provides a balanced measure when both missed pulmonary embolism cases and false positive predictions need to be considered. In addition, in order to further measure the overall classification performance of the model under different decision thresholds, this study also adopted AUC as a supplementary metric. Let the true positive rate and false positive rate be denoted by TPR and FPR, respectively, then as shown in Equation 20:


$$\begin{array}{l} \text{TPR}=\frac{TP}{TP+FN},\;\text{FPR}=\frac{FP}{FP+TN} \end{array}$$ (20)

On this basis, AUC can be expressed as the area under the ROC curve, namely as shown in Equation 21:


$$\begin{array}{l} \text{AUC}=\int_{0}^{1}\text{TPR}\left(\text{FPR}\right)d\left(\text{FPR}\right) \end{array}$$ (21)

The closer the AUC value is to 1, the stronger the model's ability to distinguish normal samples from pulmonary embolism samples under different thresholds. In contrast, if the AUC is close to 0.5, it indicates that the classification performance of the model is close to the level of random discrimination. Therefore, through the above six evaluation metrics, this study systematically assessed the performance of the proposed model on the pulmonary embolism CT recognition task from multiple perspectives, including overall prediction accuracy, reliability of positive prediction, ability to detect pulmonary embolism cases, normal case recognition ability, balance between Precision and Recall, and threshold-independent discriminative performance.

### 2.5 Experimental setup

The experiments in this study were conducted under a unified software and hardware environment to ensure the reproducibility of pulmonary embolism recognition results from CT images and the fairness of comparative analysis. Specifically, model training and testing were both performed on a deep learning platform equipped with a high performance graphics processing unit. The operating system was Ubuntu, the programming language was Python, and the deep learning framework was PyTorch, with CUDA and cuDNN used to accelerate computation. During training, the input images were first resized to a unified resolution according to the network requirements and then fed into the model. The Adam optimizer was adopted, the initial learning rate was set to 1 × 10^−4^, the batch size was set to 16, and the number of training epochs was set to 200. At the same time, the cross entropy loss function was used to optimize the model parameters in an end to end manner. To improve training stability and reduce the risk of overfitting, weight decay and a dynamic learning rate adjustment strategy were applied during training, and the model parameters corresponding to the best validation performance were saved for final testing. Table 3 summarizes the main software and hardware configurations and key hyperparameter settings used in this study. These configurations together form the basis for subsequent experimental result analysis and model performance comparison.

**Table 3:** Experimental environment and main hyperparameter settings.

Item	Setting
Operating system	Ubuntu
Programming language	Python 3.10
Deep learning framework	PyTorch
CUDA version	CUDA 11.8
cuDNN version	cuDNN 8
GPU	NVIDIA RTX 4090
Video memory	24 GB
CPU	Intel Xeon
Memory	64 GB
Input image size	224 × 224
Batch size	16
Number of training epochs	200
Optimizer	Adam
Initial learning rate	1 × 10^−4^
Weight decay	1 × 10^−5^
Learning rate scheduling strategy	Cosine annealing
Loss function	Cross entropy loss

## 3 Results

### 3.1 Experimental results compared with other baseline models

To comprehensively evaluate the effectiveness and competitiveness of the proposed method on the task of pulmonary embolism recognition from CT images, this section selected multiple representative deep learning baseline models for comparative experiments. These models cover classical convolutional neural networks, vision Transformer architectures, medical image analysis models, and hybrid architecture models with strong representation ability in recent years. By conducting a systematic comparison of different methods under a unified experimental setting and performing a comprehensive analysis with evaluation metrics including Accuracy, Precision, Recall, and AUC, the performance of the proposed method can be validated from multiple perspectives, including overall classification performance, positive sample recognition ability, and model discriminative stability. This provides an experimental basis for the subsequent further analysis of the advantages of the proposed method, and the corresponding experimental results are shown in Table 4.

**Table 4:** Five-fold cross-validation comparison experiment results.

Method	Acc	Precision	Recall	Specificity	F1-score	AUC
ResNet50	0.868 ± 0.031	0.851 ± 0.024	0.879 ± 0.037	0.858 ± 0.068	0.865 ± 0.022	0.907 ± 0.019
ConvNext	0.909 ± 0.026	0.896 ± 0.034	0.902 ± 0.021	0.915 ± 0.053	0.899 ± 0.020	0.938 ± 0.028
ResNext	0.891 ± 0.035	0.884 ± 0.027	0.897 ± 0.032	0.886 ± 0.073	0.890 ± 0.021	0.925 ± 0.023
Medmamba	0.921 ± 0.029	0.913 ± 0.018	0.911 ± 0.036	0.930 ± 0.065	0.912 ± 0.020	0.949 ± 0.026
Swin-Transformer	0.934 ± 0.033	0.928 ± 0.030	0.922 ± 0.025	0.945 ± 0.067	0.925 ± 0.020	0.957 ± 0.021
Diffmic-V2	0.926 ± 0.022	0.918 ± 0.039	0.924 ± 0.028	0.928 ± 0.049	0.921 ± 0.024	0.954 ± 0.031
EFFResNet-ViT	0.941 ± 0.037	0.948 ± 0.026	0.925 ± 0.034	0.956 ± 0.077	0.936 ± 0.022	0.965 ± 0.017
Medkan	0.902 ± 0.028	0.891 ± 0.041	0.886 ± 0.030	0.917 ± 0.060	0.888 ± 0.025	0.932 ± 0.027
Conv-SdMLPMixer	0.916 ± 0.040	0.906 ± 0.023	0.914 ± 0.035	0.918 ± 0.083	0.910 ± 0.021	0.944 ± 0.029
Proto-Caps	0.886 ± 0.034	0.878 ± 0.032	0.891 ± 0.026	0.881 ± 0.069	0.884 ± 0.021	0.921 ± 0.036
Pcdal	0.947 ± 0.025	0.936 ± 0.038	0.941 ± 0.022	0.952 ± 0.052	0.938 ± 0.022	0.968 ± 0.024
Ours	0.956 ± 0.030	0.961 ± 0.027	0.951 ± 0.033	0.961 ± 0.065	0.953 ± 0.021	0.971 ± 0.020

The comparison results show that the proposed method achieved the highest overall performance among all evaluated models across six metrics, with Accuracy, Precision, Recall, Specificity, F1-score, and AUC values of 0.956 ± 0.030, 0.961 ± 0.027, 0.951 ± 0.033, 0.961 ± 0.065, 0.953 ± 0.021, and 0.971 ± 0.020, respectively. These results indicate that the proposed method provides more balanced recognition performance for both normal and pulmonary embolism cases, while maintaining stable threshold-independent classification behavior. Compared with classical convolutional networks such as ResNet50, ResNext, and ConvNext, the proposed method better captures embolism-related imaging characteristics associated with local filling defects, intravascular density variations, vascular morphology, and contextual anatomical structures. Compared with models emphasizing global representation or hybrid feature modeling, such as Swin Transformer, Medmamba, and EFFResNet ViT, the proposed model further improves the recognition of subtle pulmonary embolism-related patterns in CT images. This improvement is mainly attributed to the Pulmonary Embolus Feature Enhancement Module and the Vascular Boundary Aware Fusion Module. The former enhances cross-level lesion-related feature interaction, whereas the latter incorporates boundary-related structural and texture cues at the feature level. As a result, the model can generate more informative representations for distinguishing pulmonary embolism-related abnormal vascular patterns from normal pulmonary vascular structures.

From a medical perspective, pulmonary embolism is an acute and severe disease with a high risk of adverse outcomes, and its early recognition is of great value for timely clinical assessment and treatment planning. The results in the table indicate that the proposed method not only achieves high overall accuracy, but also shows favorable performance on Recall and AUC, which means that while maintaining stable overall classification performance, the model can more effectively identify pulmonary embolism cases within the binary classification setting and maintain stable threshold-independent separation between normal and pulmonary embolism samples under different decision thresholds. For computer-aided screening applications, such performance characteristics are potentially beneficial for reducing classification errors in suspected pulmonary embolism cases, improving the screening efficiency for suspected pulmonary embolism patients, and providing auxiliary reference information for radiologists in diagnostic decision making. Especially in cases where imaging manifestations are complex or local density changes are subtle, a model with stronger feature-level structural representation ability is more likely to improve the practicality of an automatic recognition system, thereby providing auxiliary quantitative evidence for computer-aided pulmonary embolism screening rather than replacing clinical diagnosis.

**Figure 4:**
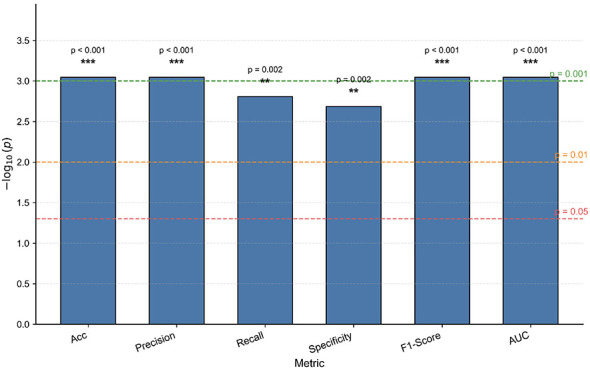
Statistical significance analysis between the proposed method and Pcdal across six evaluation metrics, where all metrics show significant differences under the paired statistical test. ^**^ denotes p < 0.01, and ^***^ denotes p < 0.001, indicating different levels of statistical significance under the paired statistical test.

Furthermore, we selected the optimal model from the comparison models and performed a statistical significance analysis, as shown in Figure 4.

The statistical significance results indicate that the proposed method achieves statistically significant improvements over Pcdal across all evaluated metrics. Specifically, Acc, Precision, F1-Score, and AUC reach the strongest significance level with *p* < 0.001, while Recall and Specificity also show significant differences with *p* = 0.002. These results suggest that the performance gains of the proposed method are not limited to numerical improvements in the mean values, but also exhibit statistical reliability across five-fold validation. Therefore, the proposed method demonstrates more stable and robust classification advantages over the strongest baseline model in terms of overall accuracy, positive-case recognition, negative-case discrimination, balanced classification performance, and threshold-independent discriminative ability.

### 3.2 Ablation experiment results

To further verify the actual contribution of each key component in the proposed method and to analyze the role of different modules in the task of pulmonary embolism recognition from CT images, this section conducts ablation experiments, and the corresponding results are shown in Table 5. The ablation design is centered on the core functional modules in the overall framework as well as the key internal branches within these modules. By progressively removing or replacing the corresponding components under a unified experimental setting, the changes in model performance are systematically compared. Based on this experimental design, the internal working mechanism of the proposed method can be evaluated in greater depth from the perspectives of overall structural effectiveness, module collaboration, and fine grained functional allocation.

**Table 5:** Five-fold cross-validation ablation experiment results.

Method	Acc	Precision	Recall	Specificity	F1-score	AUC
Backbone only	0.908 ± 0.034	0.897 ± 0.029	0.886 ± 0.037	0.928 ± 0.073	0.891 ± 0.024	0.936 ± 0.025
w/o Pulmonary Embolus Feature Enhancement Module	0.942 ± 0.026	0.934 ± 0.033	0.926 ± 0.028	0.957 ± 0.056	0.930 ± 0.022	0.966 ± 0.022
w/o Vascular Boundary-Aware Fusion Module	0.949 ± 0.031	0.943 ± 0.024	0.936 ± 0.035	0.961 ± 0.067	0.939 ± 0.021	0.969 ± 0.019
w/o feature interaction in PEFEM	0.934 ± 0.038	0.926 ± 0.027	0.918 ± 0.031	0.949 ± 0.078	0.922 ± 0.021	0.962 ± 0.029
w/o structure branch in VBAFM	0.946 ± 0.023	0.939 ± 0.036	0.932 ± 0.026	0.959 ± 0.050	0.935 ± 0.022	0.968 ± 0.027
w/o style branch in VBAFM	0.944 ± 0.035	0.938 ± 0.021	0.929 ± 0.034	0.958 ± 0.074	0.933 ± 0.020	0.967 ± 0.024
Ours	0.956 ± 0.030	0.961 ± 0.027	0.951 ± 0.033	0.961 ± 0.065	0.953 ± 0.021	0.971 ± 0.020

The ablation results show that the complete model achieved the highest values across all six metrics, including Acc, Precision, Recall, Specificity, F1-score, and AUC, indicating that the two proposed modules and their internal components consistently contributed to pulmonary embolism recognition on CT images. When only the backbone network was retained, the model performance decreased noticeably, suggesting that basic feature extraction alone was insufficient to represent subtle embolism-related findings such as local filling defects, intravascular density changes, vascular boundary variations, and surrounding anatomical context. After removing the Pulmonary Embolus Feature Enhancement Module or the feature interaction mechanism within it, the performance declined further, indicating that cross-level feature interaction is important for integrating lesion-related responses distributed across different semantic layers. After removing the Vascular Boundary Aware Fusion Module, the structural branch, or the style branch, the model also showed different degrees of performance degradation, suggesting that feature-level structural encoding and local texture reconstruction help characterize blurred vessel boundaries, small embolic regions, and low-contrast abnormal density patterns. From a medical perspective, pulmonary embolism findings on CT images can be subtle and heterogeneous, especially when emboli are small, irregularly distributed, or visually close to normal vascular structures. By enhancing lesion-related representations and incorporating boundary-related structural cues, the complete model improved positive-case recognition while maintaining balanced classification performance for normal and pulmonary embolism samples. These results support the contribution of the proposed modules to CT-based computer-aided pulmonary embolism screening under the binary classification setting.

### 3.3 Visualize experimental results

#### 3.3.1 t-SNE experimental results

**Figure 5:**
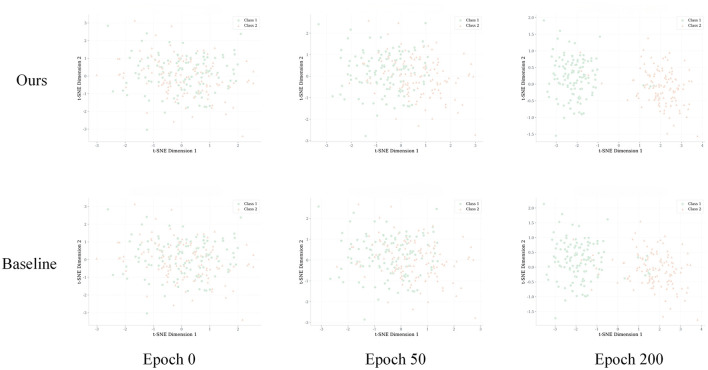
The t-SNE visualization illustrates the feature distribution evolution of the proposed method and the baseline model at Epoch 0, Epoch 50, and Epoch 200, showing that the proposed method progressively learns more compact intra-class representations and clearer inter-class separation during training.

To further analyze the representation learning process of the model from the perspective of feature space distribution, this section employs the t SNE visualization method to project and display feature representations at different training stages. By comparing the distribution changes of the proposed method and the baseline model during the feature evolution process, the modeling ability of the model with respect to category structure, intra class aggregation trends, and inter class boundary formation can be observed more intuitively. This visual analysis provides important support for understanding the formation mechanism of the internal discriminative representations of the model and its optimization process, and the corresponding results are shown in Figure 5.

The t SNE visualization results in the figure show that, at the initial stage of training, the distributions of the two classes for both the proposed method and the baseline model exhibit a clear degree of overlap, which indicates that the discriminability of the feature representations is still limited at this stage. As training proceeds to Epoch 50, the two classes corresponding to the proposed method begin to show a clearer separation trend, and the degree of intra class sample aggregation is also gradually enhanced, whereas although the baseline model also exhibits a certain degree of structural adjustment, a considerable amount of overlap still remains overall. When training reaches Epoch 200, the proposed method has formed a relatively more compact intra class distribution and a more observable inter class boundary, indicating that the model is able to learn more separable deep feature representations for pulmonary embolism classification. In contrast, the baseline model still retains a certain degree of inter class overlap and distribution dispersion at the final stage. This suggests that the proposed method has certain advantages in feature space organization ability, category separability modeling, and representation optimization stability during training, and provides qualitative support for the effectiveness of the proposed modules in enhancing the learning of pulmonary embolism related discriminative features.

#### 3.3.2 Confusion matrix experimental results

**Figure 6:**
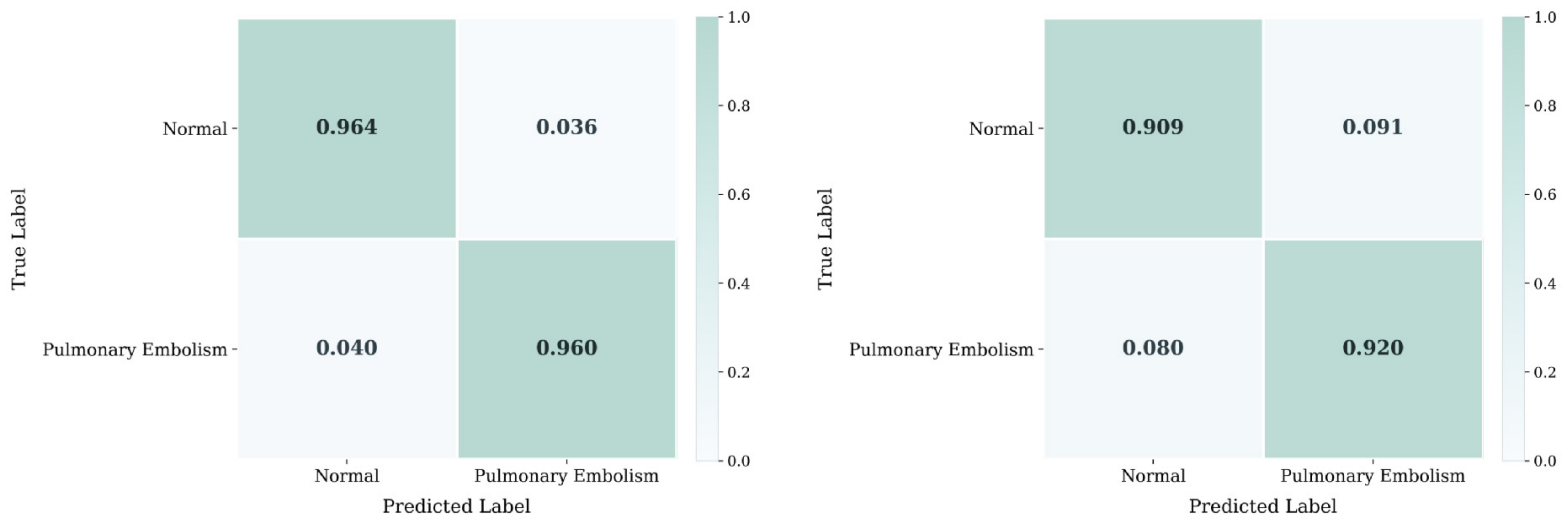
The confusion matrix presents the classification distributions of the proposed method and the baseline model for normal and pulmonary embolism samples, providing an intuitive comparison of their category-level prediction behaviors.

To further analyze the discriminative behavior of the model across different categories, this section adopts the confusion matrix to visualize the prediction results, and the corresponding results are shown in Figure 6. This form of analysis can more intuitively reflect the classification characteristics and error distribution of the model in the task of pulmonary embolism recognition from CT images by examining the correspondence between the true categories and the predicted categories.

The confusion matrix results show that the proposed method exhibits higher classification consistency in both the Normal and Pulmonary Embolism categories, as the values in the main diagonal region are significantly higher than those of the baseline model, whereas the misclassification proportions in the off diagonal region are relatively lower. This indicates that the proposed method can distinguish more accurately between normal samples and pulmonary embolism samples. Specifically, the correct recognition rate of the proposed method reaches 0.964 for normal samples and 0.960 for pulmonary embolism samples, both of which are superior to the corresponding values of 0.909 and 0.920 achieved by the baseline model. At the same time, the proportions of misclassifying normal samples as pulmonary embolism and misclassifying pulmonary embolism as normal are controlled at 0.036 and 0.040, respectively, showing a more balanced dual category recognition ability. This demonstrates that the proposed model not only has advantages in overall classification performance, but also performs better in reducing the risks of missed diagnosis and misdiagnosis. Especially for pulmonary embolism, which is a clinically high risk disease, stronger positive recognition ability and a lower misclassification rate are of great significance for improving the efficiency of aided screening and the reliability of clinical diagnosis.

#### 3.3.3 Vascular boundary-aware visualization results

**Figure 7:**
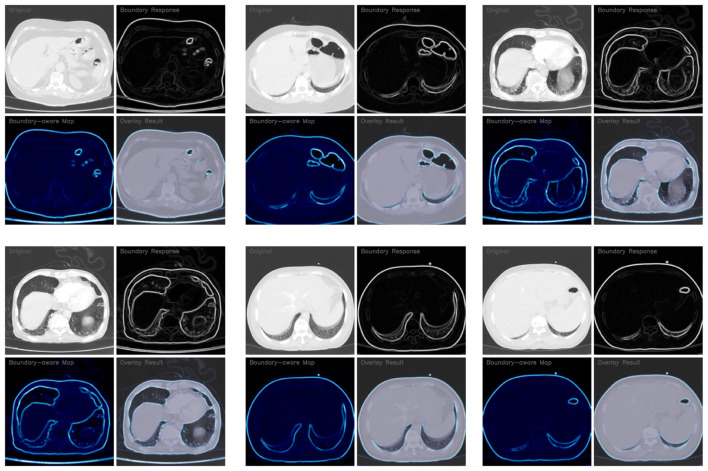
Visualization results of vascular boundary-aware representations on representative CT images, including the original image, boundary response, boundary-aware map, and overlay result, showing how the model captures structurally relevant boundary information in pulmonary embolism CT analysis.

To further analyze the model's perception process of key structural information from the perspective of interpretability, this section provides a visual presentation of vascular boundary related responses. This part is mainly used to observe the model's attention to structural contours, local transition regions, and boundary related semantic cues during the feature learning process on CT images. Through the joint presentation of the original image, boundary response map, boundary aware map, and their overlay results, the functional manner of the proposed boundary aware mechanism in feature modeling can be understood more intuitively, and the corresponding results are shown in Figure 7.

The visualization results show that the boundary response map can clearly highlight structurally meaningful regions such as the thoracic contour, lung boundaries, and the interfaces between vessels and surrounding tissues. In the boundary aware map, these locations further form more continuous and more concentrated high response distributions, which indicates that the model does not rely only on coarse global grayscale differences for discrimination, but instead performs targeted modeling of boundary information related to anatomical structural changes. In the overlay results, the boundary aware regions remain well aligned with the important anatomical contours in the original CT image, which demonstrates that the proposed Vascular Boundary Aware Fusion Module can effectively enhance the representation ability for local structural changes and boundary transition regions. This is of great significance for recognizing complex imaging patterns in pulmonary embolism tasks where boundaries are blurred, density changes are subtle, or local abnormalities are not obvious, and it also verifies the rationality of the module design from the perspective of interpretability.

### 3.4 Results of off-domain validation experiments

To further evaluate the robustness and cross-domain transferability of the proposed method, this study used the single-center dataset as the source domain and introduced two public datasets, FUMPE and RSNA, as independent target domains for external validation. For the external datasets, all externally available labeled samples were retained after basic data availability checking. Samples were excluded only when labels were missing, image files were incomplete, or the images could not be successfully processed for pulmonary artery assessment. The positive and negative categories were defined according to the original annotations or diagnostic labels provided by each public dataset. Since different datasets may vary in case composition, scanning devices, imaging protocols, image quality, and annotation criteria, evaluation on unseen external data provides a more realistic assessment of model adaptability under domain shift. During external validation, FUMPE and RSNA were processed using the same preprocessing pipeline as the internal dataset, including image format unification, size standardization, grayscale normalization, label alignment, and dataset organization. No external samples were used for model training, parameter optimization, or threshold recalibration, and the classification threshold determined from the internal validation process was directly applied to the external datasets. Through this source-to-target validation setting, the generalization performance of the proposed method was assessed under cross-center and cross-distribution conditions, and the corresponding results are shown in Table 6.

**Table 6:** External validation results on the FUMPE and RSNA datasets.

Dataset	Method	Acc	Precision	Recall	Specificity	F1-score	AUC
FUMPE	ResNet50	0.688 ± 0.041	0.681 ± 0.034	0.659 ± 0.046	0.715 ± 0.051	0.670 ± 0.035	0.735 ± 0.029
	ConvNext	0.715 ± 0.037	0.704 ± 0.043	0.692 ± 0.031	0.736 ± 0.046	0.698 ± 0.034	0.751 ± 0.036
	Swin-Transformer	0.738 ± 0.032	0.746 ± 0.039	0.709 ± 0.044	0.765 ± 0.052	0.727 ± 0.037	0.772 ± 0.027
	Diffmic-V2	0.751 ± 0.045	0.728 ± 0.035	0.759 ± 0.038	0.744 ± 0.055	0.743 ± 0.034	0.784 ± 0.041
	Pcdal	0.762 ± 0.034	0.756 ± 0.047	0.744 ± 0.030	0.778 ± 0.043	0.750 ± 0.035	0.796 ± 0.033
	Ours	0.779 ± 0.039	0.772 ± 0.036	0.761 ± 0.043	0.795 ± 0.049	0.766 ± 0.038	0.815 ± 0.028
RSNA	ResNet50	0.609 ± 0.044	0.605 ± 0.038	0.581 ± 0.049	0.635 ± 0.054	0.593 ± 0.041	0.671 ± 0.034
	ConvNext	0.607 ± 0.036	0.617 ± 0.045	0.568 ± 0.041	0.643 ± 0.048	0.591 ± 0.039	0.662 ± 0.030
	Swin-Transformer	0.636 ± 0.041	0.622 ± 0.033	0.614 ± 0.046	0.656 ± 0.052	0.618 ± 0.038	0.687 ± 0.037
	Diffmic-V2	0.644 ± 0.038	0.635 ± 0.042	0.645 ± 0.035	0.643 ± 0.047	0.640 ± 0.036	0.694 ± 0.045
	Pcdal	0.655 ± 0.047	0.650 ± 0.031	0.632 ± 0.044	0.676 ± 0.055	0.641 ± 0.037	0.707 ± 0.036
	Ours	0.672 ± 0.040	0.665 ± 0.037	0.654 ± 0.048	0.688 ± 0.050	0.659 ± 0.041	0.725 ± 0.032

The external validation results show that the overall performance of all methods on the two public datasets, FUMPE and RSNA, decreased compared with the internal experiments, which indicates that distribution differences, imaging protocol differences, and sample complexity under cross source data conditions impose higher requirements on model generalization ability. However, the proposed method still maintained the best performance on both external datasets among the compared models, although its absolute performance also showed an evident decline compared with the internal test results. On the FUMPE dataset, the proposed method achieved Acc, Precision, Recall, and AUC values of 0.779 ± 0.039, 0.772 ± 0.036, 0.761 ± 0.043, and 0.815 ± 0.028, respectively, which were overall superior to those of the other comparison models, indicating that the proposed method retained a certain degree of discriminative ability under moderate domain shift. On the more complex RSNA dataset, the metrics of all models declined further, and the proposed method achieved Acc, Precision, Recall, and AUC values of 0.672 ± 0.040, 0.665 ± 0.037, 0.654 ± 0.048, and 0.725 ± 0.032, respectively. This result suggests that stronger domain discrepancy still substantially affects the recognition performance of the model, even though the proposed method remained relatively competitive compared with the baseline methods.

The decrease in external validation performance may be related to several factors. First, the FUMPE and RSNA datasets differ from the single center dataset used in this study in terms of imaging protocols, scanner settings, reconstruction parameters, contrast enhancement characteristics, and image quality. Second, differences in case composition, disease severity, embolism location, and annotation or label definitions may lead to distribution shifts between the source and target domains. Third, the classification threshold optimized on the internal dataset may not be directly transferable to external datasets with different data distributions, which may further affect Accuracy, Precision, and Recall. Therefore, the external validation results should be interpreted as evidence that the proposed method has preliminary cross-domain transfer potential, rather than as sufficient proof of robust clinical generalization. More comprehensive multi-center validation, domain adaptation, and threshold calibration are still required before broader clinical application.

### 3.5 The impact of learning rate on experimental results

**Figure 8:**
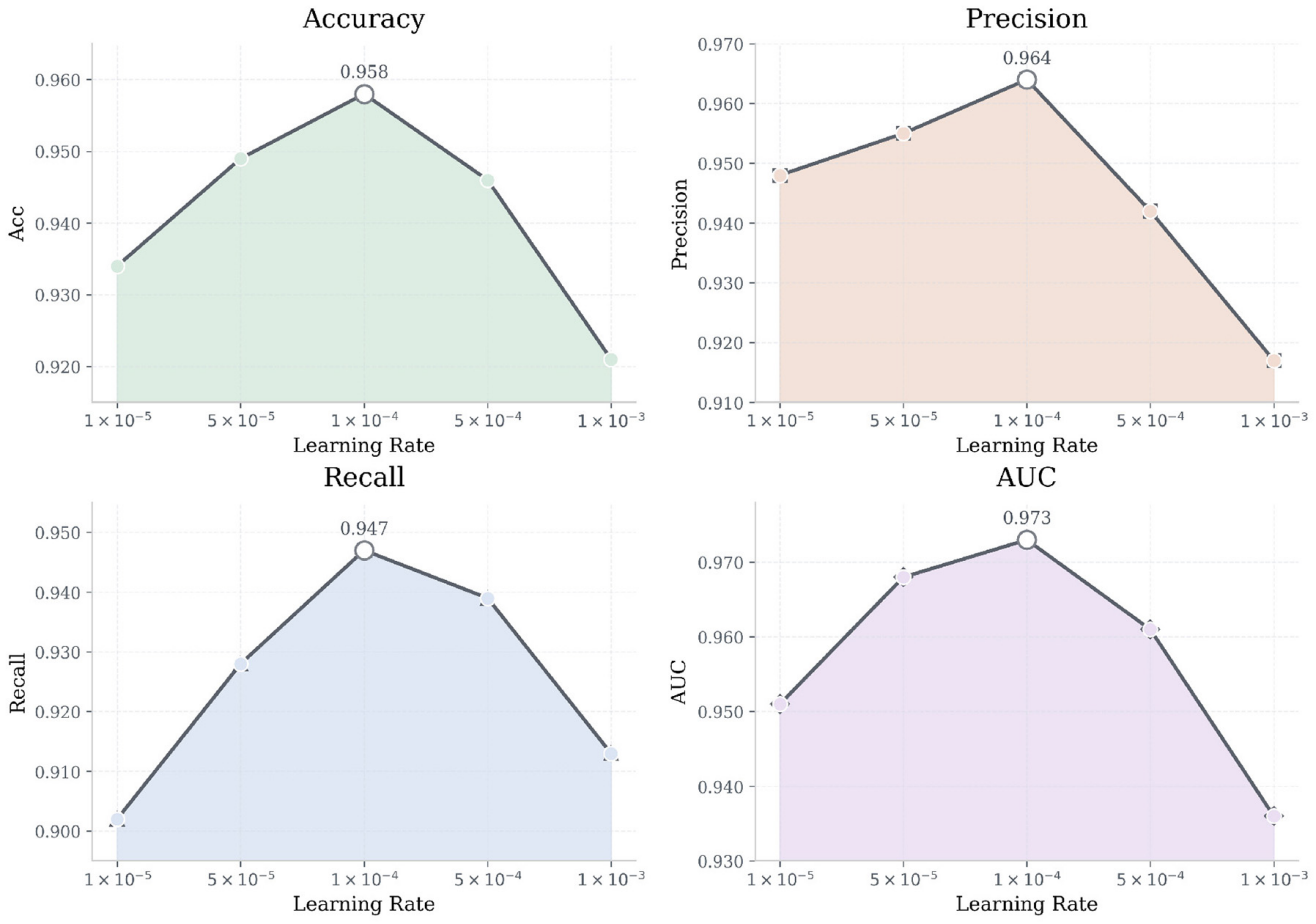
The impact of learning rate on experimental results.

To further analyze the influence of key optimization parameters on model performance during training, this section conducts a hyperparameter sensitivity experiment centered on the learning rate setting. By comparing the performance variations corresponding to different learning rates under a unified experimental condition, the convergence characteristics and parameter stability of the model during the optimization process can be evaluated more intuitively, and the corresponding results are shown in Figure 8. Specifically, the results presented in this sensitivity analysis were obtained from one representative fold selected from the five-fold cross-validation experiments.

It can be seen from the figure that the learning rate has a significant influence on model performance, and all four evaluation metrics generally exhibit a trend of first increasing and then decreasing, which indicates that either an excessively small or an excessively large learning rate can adversely affect the optimization performance of the model. When the learning rate increases from 1 × 10^−5^ to 1 × 10^−4^, Accuracy, Precision, Recall, and AUC all gradually improve and reach their optimal values of 0.958, 0.964, 0.947, and 0.973, respectively, at 1 × 10^−4^. This indicates that the model achieves a better balance between parameter update step size and convergence stability at this stage, and is therefore able to learn pulmonary embolism related discriminative features more effectively. However, when the learning rate is further increased to 5 × 10^−4^ and 1 × 10^−3^, all metrics decline to different degrees, and the decrease becomes more obvious at 1 × 10^−3^, which suggests that an excessively large learning rate may intensify oscillations during the optimization process and weaken the model's ability to effectively characterize key structural information and lesion related features. Overall, 1 × 10^−4^ is a more appropriate learning rate setting in this study, and this further verifies that the model can achieve more stable and superior recognition performance under a reasonable optimization parameter configuration.

## 4 Discussion

Although the proposed method achieved favorable performance in the task of pulmonary embolism recognition from CT images, several limitations still deserve further attention. First, the data used in this study were still mainly derived from single-center retrospective data. Although external validation was conducted by introducing public datasets, complex differences often exist across different centers in terms of scanning devices, imaging protocols, case composition, and annotation standards. Such distribution shifts may still affect model stability and generalization ability, and therefore the current results cannot fully represent the applicability of the model in broader real-world clinical environments (17, 18). Second, the imaging manifestations of pulmonary embolism are highly heterogeneous. Especially when lesions are small in extent, have blurred boundaries, or are accompanied by other pulmonary abnormalities, the model may still face considerable difficulty in fine-grained discrimination. In addition, although this study improved the modeling ability of the model for key structural information through feature enhancement and a boundary-aware mechanism, from the perspective of clinical interpretation, how to further establish more stable, transparent, and fully understandable discriminative evidence for physicians remains an important direction for future improvement.

In terms of clinical performance interpretation, the current study has expanded the evaluation metrics to include Accuracy, Precision, Recall, Specificity, F1-score, and AUC, and the confusion matrix was further introduced to describe category-level prediction behavior. In the binary classification setting, Recall corresponds to Sensitivity, and Precision corresponds to the positive predictive value, which enables the model to be evaluated from the perspectives of positive-case detection, false-positive control, normal-case recognition, balanced classification performance, and threshold-independent discrimination. Nevertheless, several clinically important analyses remain insufficiently explored in the present study. For example, negative predictive value, false-negative burden, false-positive burden, calibration performance, decision-curve analysis, and threshold-dependent sensitivity-specificity trade-offs were not fully investigated. This is mainly because the current work was based on retrospective image-level binary classification using representative two-dimensional CT images, while the external datasets also differed in label definitions and data organization. Therefore, future studies should further perform patient-level validation, calibration analysis, decision-curve analysis, and detailed false-negative case review under larger multi-center clinical settings, so as to more comprehensively evaluate the clinical utility and deployment reliability of the proposed method.

From the perspective of clinical application, artificial intelligence-based automatic pulmonary embolism recognition methods have considerable practical translational potential. Pulmonary embolism is an acute and critical disease with rapid progression and a high risk of fatality, and its early detection and timely intervention are of great importance for patient prognosis. Artificial intelligence models can rapidly perform automatic screening and risk prompting from a large number of CT images, thereby providing effective assistance for radiologists (19, 20). Existing studies have shown that artificial intelligence methods can not only achieve relatively stable classification ability in real-world data, but can also function as triage tools, second-reading tools, or prioritization tools in clinical workflows, thereby improving the identification efficiency of suspicious cases and shortening the processing time of critical cases (21, 22). Therefore, while maintaining high recognition performance, the proposed method is expected to play a positive role in pulmonary embolism-aided diagnosis, emergency triage, and high-risk case warning if it can be further integrated with hospital imaging information systems and clinical workflows, and it may also help relieve the burden of clinical image reading and improve diagnostic consistency to some extent.

Future research can further focus on model trustworthiness, generalization, and clinical deployability. On the one hand, continuous validation should be conducted under larger-scale, multi-center, multi-device, and multi-population data conditions to systematically evaluate the robustness, fairness, and transferability of the model in complex clinical environments, and to further improve its reliability in real application scenarios (23, 24). On the other hand, future studies may further incorporate multimodal clinical information, such as laboratory indicators, vital signs, medical history information, and imaging reports, in order to construct a more comprehensive intelligent-aided diagnosis framework for pulmonary embolism. At the same time, it is also necessary to strengthen interpretability design, human-machine collaborative decision mechanisms, and standardized evaluation during deployment, so as to promote artificial intelligence methods from laboratory research toward safer, more transparent, and more clinically valuable practical applications (23, 24).

## 5 Conclusion

This study focused on the task of automatic pulmonary embolism recognition from CT images and proposed a deep learning method that combines feature enhancement and feature-level boundary aware fusion mechanisms. To address the challenges that pulmonary embolism in CT images often presents complex lesion morphology, subtle local density differences, and inconspicuous vascular boundary changes, this study designed a Pulmonary Embolus Feature Enhancement Module and a Vascular Boundary Aware Fusion Module within the overall framework. These two modules were used to strengthen cross level lesion discriminative feature modeling and enhance the semantic representation ability of boundary-related structural cues and local features, respectively. The results of comparative experiments, ablation studies, visualization analysis, and external dataset validation on the single center dataset demonstrated that the proposed method achieved favorable comprehensive performance on metrics including Accuracy, Precision, Recall, and AUC. At the same time, it also showed certain advantages in feature separability, classification stability, and cross source data generalization ability, indicating that the proposed method can provide a useful technical reference for computer-aided pulmonary embolism screening from CT images.

Future research can further focus on multi center data expansion, improvement of model trustworthiness, and validation for potential clinical workflow adaptation. On the one hand, it is necessary to continuously validate model performance on larger scale and more diverse external independent data in order to further enhance the robustness and generalization ability of the method under complex clinical scenarios. On the other hand, multimodal data such as clinical text, laboratory examination indicators, and medical history information can be incorporated to construct a more comprehensive computer-aided screening and risk assessment framework for pulmonary embolism. In addition, since the present study was formulated as a binary classification task rather than an explicit vascular boundary segmentation or lesion localization task, future studies should further incorporate pixel-level annotations, lesion localization labels, and subgroup analyses of small-scale emboli to more directly validate the boundary-related and clinical implications of the proposed method. In addition, further efforts should be made to strengthen model interpretability analysis, human machine collaboration mechanism design, and deployment evaluation in real clinical workflows. Through these efforts, it is expected that intelligent pulmonary embolism recognition methods can be promoted from the stage of experimental validation toward more rigorous clinical validation, thereby providing more reliable auxiliary evidence for computer-aided pulmonary embolism screening and assessment and improving the efficiency of early pulmonary embolism screening and the quality of computer-aided assessment.

## Data Availability

The original contributions presented in the study are included in the article/supplementary material, further inquiries can be directed to the corresponding author.
